# Medical and pharmacy students' perceptions of the grading and assessment practices

**DOI:** 10.3389/fpsyg.2013.00423

**Published:** 2013-07-10

**Authors:** C. D. Kasanda, K. H. Mitonga, K. Veii, R. F. Zimba

**Affiliations:** ^1^Department of Mathematics, Science and Sport Education, Faculty of Education, University of NamibiaKhomas, Namibia; ^2^Department of Community Medicine, FOHS, School of Medicine, University of NamibiaKhomas, Namibia; ^3^Department of Education Psychology and Inclusive Education, Faculty of Education, University of NamibiaKhomas, Namibia

**Keywords:** students' perceptions, grading, assessment practices, higher education, assessment, University students, Namibia

## Abstract

Many students at the University of Namibia have frequently complained about ineffective assessment practices used at the institution. On many occasions, these complaints have not been substantiated with evidence of any kind. The purpose of this study was to obtain some empirical evidence that would ascertain undergraduate students' perceptions of the University of Namibia's grading and assessment practices. Using a structured scaled questionnaire, data were obtained from a representative sample of the University's undergraduate students studying for Medical and Pharmacy degrees. The questionnaire items covered matters related to students' experiences of assessment practices, feedback on assessment tasks, reliability and validity of assessment tools used by lecturers, efficacy of processes of administering examinations, perceptions of irregular and unfair assessment practices, impact of assessment regimes on students' cost of studies, motivation, morale, rate of progression in studies and graduation, the degree of compliance with assessment ethics and on academic quality assurance. According to the data reported in this article, the majority of the respondents perceived that the Schools of Medicine and Pharmacy at the University of Namibia applied assessment practices that yielded reliable and valid results. This was the case because most lecturers in the two schools used appropriate assessment tools and provided their students with prompt and informative feedback on the results of assignments, tests and examinations. In addition, most respondents reported that whereas examination procedures used in the two schools were efficient and effective, lecturers graded examination scripts fairly. These and other results are discussed in the article to communicate the message that the assessment procedures used in the Schools of Medicine and Pharmacy at the University of Namibia would promote effective learning and understanding amongst students as they were of high quality.

## Introduction and background

Assessment is an important and essential component of any general education and higher education in particular (McMillan et al., 2002; Saliu, 2005). According to Airasian (1997, p. 3), assessment is a “…process of collecting, synthesizing, and interpreting information to aid in decision making….” It is essential in improving the teaching process, subject content offered and understanding, its delivery and service delivery to the students so as to ensure that the graduate of such a system is of high quality and is able to effectively carry out assigned tasks in the work place.

When defined in this way, assessment is not merely concerned with testing or measurement. It is a process that enables University staff make judgments about the value of student achievement. It certifies and makes available information communicating that intended standards of achievement, skill and performance have been attained by students (Zimba, 2005). When understood in this way, assessment confounds the criticism of its value which is contained in the saying that “pigs are not fattened by being weighed” (Brown and Knight, 1994). Instead, its main purpose is that of ascertaining and monitoring student achievement, development and well-being. Through this, assessment either questions or guarantees the quality of University graduates. We use the term *assessment* in this article to refer to a variety of procedures that are employed to promote, ensure and ascertain levels of and quality of learning and performance demonstrated by students in programmes of instruction. These include observations, ratings of performance, paper and pencil tests and examinations, evaluations of all sorts, scaled measurements, etc. (Miller et al., 2012).

The main issue we intended to investigate in this study was to ascertain whether the assessment procedures and practices used by members of staff in the Schools of Medicine and Pharmacy at the University of Namibia were consistent with this understanding of assessment.

The University of Namibia (UNAM, 2011, p. 12, 20) like any other institution of higher education ensures that the academic achievement of its students taking any module or course is reliably and validly assessed. This assessment at UNAM takes the form of either continuous assessment through assignments, tests, presentations, projects, etc. or final examinations. The final examination may last from one and a half to three hours depending on the credits carried and content covered.

The University also uses the system of external examiners to underwrite the quality of its assessment processes. The external examiners come from other Universities within and in some cases beyond the Southern African Development Community (SADC) region, who act as quality assurance agents to the courses offered (Njabili and Kasanda, 2005). In this way the university's offerings could be said to be comparable to offerings in other institutions of similar standing.

As summative assessment mechanisms, and for them to guarantee certificates that students receive, end of semester or end of year examinations should be conducted in a reliable and valid manner. For this to happen, examination questions should be of high quality, there should be institutional security for examination papers, examinations should be invigilated adequately to avoid cheating, examination scripts should be critically and carefully marked, external examiners should be given sufficient time to moderate the marking of the scripts, there should be security of examination marks to avoid fraud and that these marks should be meticulously processed by examination boards. When these processes and procedures are not adequately followed, the integrity of the assessment process is compromised and the certificates that students receive become valueless (Zimba, 2005). One of the purposes of this study was to find out whether the examination processes and practices used by the School of Medicine and the School of Pharmacy staff at the University of Namibia adhered to high quality assessment standards.

Reform of assessment methods and regimes is currently a concern in many countries around the world (Morgan and Watson, 2002). There is much discussion about the design of assessment tasks and systems that will provide valid and useful information for making a variety of educational decisions, including provision of feedback to teachers and students to large-scale monitoring and evaluation of educational systems (Morgan and Watson, 2002).

The main reasons for the assessment of students at all levels of education can be divided into two categories: The formative assessment whereby the examiner or teacher assesses the knowledge about the topic, the motivation level, the understanding of main points, the class preparation and the problem students may have with the learning materials.

The summative assessment is usually implemented at the end of a unit or course with the aim of enabling the instructor to ascertain the extent to which instructional objectives were attained. Generally, this kind of assessment does not benefit the teacher and the learners in the sense that the students' shortcomings cannot be used to improve their performance or the teacher's teaching strategies. The students often than not move on to the next Grade or year of study or enter a Institution of Higher Education depending on their performance on the summative assessment.

Generally, the learning objectives and the purpose of the assessment should guide the examiner or teacher in the selection and design of assessment. Thus when assessment and learning objectives are aligned, better student performance is most likely to occur.

## Purpose of the study

The purpose of this study was to ascertain perceptions of University of Namibia's medical and pharmacy students regarding assessment procedures, practices, quality of assessment tools and assessment ethical considerations taken during the formative and summative assessment periods. The main goal of the study was to find out from the students their perceived evaluation of the quality of the assessment regimes applied by lecturers in the Schools of Medicine and Pharmacy at the University of Namibia. Specifically, the study sought students' perceptions on:

- General assessment practices used by lecturers in the Schools of Medicine and Pharmacy;
- Provision of feedback to students on assignment and test results;
- Administration and conduct of examinations;
- Fairness of assessment practices;
- Impact of assessment regimes on student morale, study progression, graduation and study costs;
- Ethical conduct of assessments.

## Materials and methods

### Sample

A convenient sample of 148 first and second year medical and pharmacy students at the University of Namibia acted as the source of data for the study. The mean age for the 148 students was 21.84 years. Twenty nine (19.6%) of the respondents were male and 119 (80.4%) were female; 97 (65.5%) were first year students and 51 (34.5%) were second year students.

### Research instrument

A structured scaled 54-item questionnaire was used to collect data from the sample. The questionnaire comprised mainly close-ended questions. In order to find out whether the questionnaire items measured the same concept, Cronbach's alpha for internal consistency was calculated and found to be 0.828. Tavakol and Dennick (2011, p. 53), note that “Internal consistency describes the extent to which all the items in a test measure the same construct and hence it is connected to the inter-relatedness of the items within the test”. The high Cronbach's alpha in our study shows that the questionnaire was reliable (Santos, 1999).

The questions on the questionnaire focused on the following areas: (1) demographic data of the respondents; (2) Practices and experiences of grading and assessment; (3) Perceptions on continuous assessment feedback; (4) Perceptions on Final examination; (5) opinions regarding the administration of the examinations; (6) Views on fairness of examinations; (7) Assessment and grading impact on motivation and/or morale, progression, graduation and expenses; (8) Ethical conduct of examiners; (9) Quality assurance—internal and external moderation.

### Procedure

The questionnaire was administered to respondents by the researchers. The respondents were required to rate their agreement or disagreement on each statement by choosing one of the following responses: Strongly Agree (1), Agree (2), Disagree (3) or Strongly Disagree (4).Data were analysed using the Fisher exact tests for dependent proportions.

### Data analysis

Proportions were used to summarize the characteristics of the respondents because the data consisted of categorical variables. The mean scores and 95% confidence intervals were calculated for each item. In addition, associations between the respondents' baseline characteristics and their attitudes and perceptions regarding the grading and assessment practices were compared using the Chi-square test. For comparing across groups the Fisher Exact Test was used. For all statistical tests, a value of *p* < 0.05 was used to test for significance. The SPSS software program version 20 was used in the analysis of the data.

### Ethical considerations

Approval for the conduct of the study was granted by the Research Ethics Committee of the University of Namibia. The respondents were informed of their right not to take part in the study if they so wished or to withdraw from it at any point during the data collection process.

## Results and their discussion

In general, it was found that the male and female students did not significantly differ in their views and perceptions related to assessment practices and experiences (*p* > 0.05) using the Fisher Exact Test and the Pearson Chi-Square Test. Also the year of study did not seem to affect the students' views and perceptions of the assessment practices at UNAM. The year 1 and year 2 students were not statistically different in their views and perceptions agreement/disagreement in most of the items (See Tables 1–7). The rest of the results are presented as follows.

**Table 1:** **Experiences and practice of assessment at the University of Namibia as perceived by the respondents**.

	***N***	**Minimum**	**Maximum**	**Score**		**95% confidence interval**
				**Mean**	**Std. error**	
My lecturers give at least two assignments in the Modules I take	148	1	2	1.22	0.034	1.5–1.28
My lecturers provide clear instructions on how to do the assignments	148	1	2	1.89	0.026	1.74–1.94
My lecturers give clear instructions on tests	148	1	2	1.89	0.026	1.74–1.94
My lecturers give unannounced tests	148	1	4	2.55	0.078	2.40–2.70
My lecturers give the scope of the work to be covered on the examinations	148	1	3	1.92	0.062	1.80–2.04
The duration of examinations should vary according to the content covered in a module	148	1	4	2.00	0.077	1.85–2.15
My examination timetable is usually too crowded (e.g., writing 2 examination papers in a day)	148	1	4	2.64	0.087	2.47–2.81
There are usually few invigilators during examinations	148	1	4	2.76	0.076	2.61–2.91
Sometimes wrong examination papers have been administered to students	148	2	4	3.45	0.056	3.34–3.56
Subject lecturers often do not turn up during the first 15 min of the examinations to answer student questions	148	1	4	2.97	0.087	2.80–3.14
Lecturers do not penalize late submission of assessment tasks	148	1	4	3.00	0.087	2.83–3.17
Some examination papers are too easy to assess learning outcomes at degree level	148	2	4	2.78	0.065	2.65–2.91
Most examination papers only test factual knowledge and not student understanding	148	1	4	3.45	0.056	3.34–3.56

### Practices and experiences

The results in Table 1 present the respondents' answers to the statements asking them to indicate their experiences with the conduct of assessment by staff in the School of Medicine and the School of Pharmacy at the University of Namibia.

With regard to whether or not the lecturers gave at least two assignments in the module students took, 78.4% of the students strongly agreed and 21.6% agreed, giving a mean score of 1.22 (95% Critical Interval: 0.97–1.21). However, regarding whether lecturers provided clear instructions on how to do the assignments and the tests, 10.8% of the students strongly agreed, while 89.2% agreed with the two statements, giving a mean scores of 1.89 (95% Critical Interval: 1.88–2.04) and 1.89 (95% Critical Interval: 1.79–2.03) respectively. These two results seem to reflect the consistency with which the lecturers clarified the instructions on both assignments and the tests used for assessing the students at the University of Namibia. This further seems to suggest that the lecturers provided clear information to students of what was required of them on both assignments and tests.

The students' responses to whether the lecturers gave unannounced tests varied. About 11% of the respondents Strongly Agreed with the statement, 44.6% agreed, while 21.6% strongly disagreed and 23.0% disagreed. The mean score on this item was 2.55(95% Critical Interval: 1.52–2.40). The divergent views on this item might be attributed to the possibility that perhaps some lecturers gave unannounced tests. It is also possible that some students had no problem with lecturers administering an unannounced test. With regard to the provision of a scope of content to be covered during the examinations, the results showed that the majority of the students agreed that lecturers tended to provide such information. About 32% of the respondents strongly agreed and 43.2% agreed while 24.3% disagreed. This gives a mean score of 1.92 (95% Critical Interval: 1.64–2.28). The availability of an examination scope might have implications on the popularity of the lecturer. Lecturers who did not provide a scope for the examinations fell foul to their students who often regarded them as unhelpful or inconsiderate. While a scope of the content to be covered on an assessment might prove useful to the students in reducing the amount of work to cover during their examination preparation, it might leave them with a gap in the overall knowledge they need to learn in a particular subject, a situation that might have adverse implications in their work place upon graduation later, especially in areas such as medicine and pharmacy. An examination-driven medical and pharmaceutical training may result in poorly prepared medical doctors and pharmacists, needing further in-service training upon graduation since their results were a mere reflection of regurgitation of a small amount of content.

The duration of the examinations vary from 2 to 3 h at the University of Namibia. This variation depends on the content covered in a module or the number of credits assigned to the module. The respondents were asked to indicate whether they were in agreement with such a variation in the duration of the examinations used to assess the students' grasp of the content at UNAM. The majority of the respondents agreed (i.e., 32.4% strongly Agreed and 45.9% agreed, while 10.8% disagreed and 10.8% strongly disagreed) with the statement. The mean score for this item was 2.00 (95% Critical Interval: 1.48–2.27).

The number of modules to be written on any one given examination day is currently one of the contentious issues at the University of Namibia as far as assessment is concerned. Many students have complained regarding the writing of two or three examinations in a day. The results seem to reflect the respondents' discontent with this practice. Nonetheless, the majority of the students at the UNAM's Schools of Medicine and Pharmacy disagreed with the statement that the examination timetable was too crowded. Twenty three per cent of the respondents strongly agreed and 12.2% agreed while 43.0% disagreed and 21.6% strongly disagreed. The mean score was 2.64 (95% Critical Interval: 2.51–3.41). The students' paradoxical disagreement with this statement might be attributed to the fact that the two schools in this study have just been recently established at the University of Namibia and have a small number of students compared to the other Faculties in the University. The smaller number of students makes it possible for the examination timetable not to be crowded. As a result the students' timetable is still without the crowdedness experience at larger campuses of the University of Namibia.

According to Grunwald and Peterson (2003) getting staff members to be actively involved in institutional activities is not easy. At the University of Namibia invigilation of examinations is one of the many contentious issues. The experiences at the UNAM Schools of Medicine and Pharmacy seem to be different. This is reflected in the students' disagreement that there are usually few invigilators during examinations. The results indicated that 12.2% of the students Strongly Agreed and 21.6% agreed with the statement. However, 21.6% Strongly Disagreed and 44.6% Disagreed. The mean score for this item was 2.76 (95% Critical Interval: 2.19–3.03).

Wrong examination papers are sometimes delivered to examination venues at the University of Namibia. This creates confusion and delays in the administration of examinations for the modules involved. In some cases, examinations are rescheduled to a later date because of the confusion. To assess the prevalence of this, one of the items on the questionnaire sought to find out the students' views on this matter. The results showed that the majority of the students disagreed that wrong examination question papers have been administered to them. About 55% of the respondents Strongly Disagreed and 33.8% disagreed while only 10.8% agreed that a “wrong” paper had been administered to them during the end of semester or year module(s). The mean score of 3.45 (95% Critical Interval: 3.58–3.88) seems to confirm the students' disagreement with the statement. The new Schools of Medicine and Pharmacy seem to have been spared the “wrong” administration of the examination papers to a large extent. Again the small number of modules and numbers of papers to be examined might contribute to this state of affairs.

The University of Namibia requires its lecturers to be present at the examinations for the first 15 min in order to solve any problems students may have about the examination questions papers. To find out whether the lecturers do meet this requirement, the researchers asked the students to indicate their agreement with the statement addressing this issue. Close to 11% (10.8%) of the respondents Strongly Agreed and 24.3% Agreed. However, 21.6% Disagreed and 42.2% Strongly Disagreed. The mean score for this statement was 2.97 (95% Critical Interval: 2.54–3.38), suggesting that the majority of lecturers do show up as per University requirements.

As to whether the lecturers do not penalize late submission of assessment tasks, the results showed that 10.8% of the respondents Strongly Agreed, 23.0% Agreed, 21.6% Disagreed and 44.6% Strongly Disagreed with the statement. The mean score was 3.00 (95% Critical Interval: 2.64–3.44). Again, the results seem to suggest that lecturers do penalize students for submitting their assessment tasks later than the stipulated deadlines. This practice is deemed essential by most lecturers in order to instil a sense of responsibility and accountability in the students.

Another question of interest to the researchers was whether some examination papers were too easy to assess learning outcomes at the honors degree level. The results indicated that 44.6% Agreed, 32.4% Disagreed, and 23.0% strongly disagreed. The mean score was 2.78 (95% Critical Interval. 2.56–3.18). According to the results, the majority of the students disagreed that the examination papers were too easy to assess their learning outcome. This seems to indicate that the examination questions were at the “right” level for these respondents.

University education is about equipping students with analytical and critical thinking skills in order to be able to solve problems of any nature effectively. Hence, the students were asked to indicate whether the examinations at the University of Namibia tested factual knowledge only or their understanding of the subject matter. The results showed that 10.8% strongly Agreed, 23.0% Agreed, 21.6% Disagreed and 44.6% strongly Disagreed with the statement. The mean score was 3.45 (95% Critical Interval: 2.74–3.16). The majority (66.2%) of the students disagreed with the statement, which seems to suggest that the question papers assessed their understanding of the subject matter.

### Continuous assessment feedback

Table 2 presents the responses given by the 148 Medical and Pharmacy first and second year students to the items on the provision of feedback to students upon completion and handing in of completed tasks.

**Table 2:** **Factors associated with the students' views and perceptions on practices and experience**.

	**Gender**	**Strongly agree**	**Agree**	**Disagree**	**Strongly disagree**	**Fisher Exact Test**	**Pearson Chi-Square Test**
						***p*-value**	***p*-value**
My lecturers give at least two assignments in the Modules I take	Male	25	4	0	0	0.32	0.32
	Female	91	28	0	0		
My lecturers provide clear instructions on how to do the assignments	Male	3	26	0	0	1.00	1.00
	Female	13	106	0	0		
My lecturers give clear instructions on tests	Male	4	25	0	0	0.519	0.739
	Female	12	107	0	0		
My lecturers give unannounced tests	Male	5	8	6	10	0.073	0.088
	Female	11	58	28	22		
My lecturers give the scope of the work to be covered on the examinations	Male	8	15	6	0	0.650	0.626
	Female	40	49	30	0		
The duration of examinations should vary according to the content covered in a module	Male	14	10	3	2	0.251	0.232
	Female	34	58	13	14		
My examination timetable is usually too crowded (e.g., writing 2 examination papers in a day)	Male	4	2	12	11	0.114	0.082
	Female	30	16	52	21		
There are usually few invigilators during examinations	Male	4	6	14	5	0.932	0.917
	Female	14	26	52	27		
Sometimes wrong examination papers have been administered to students	Male	0	2	7	20	0.289	0.260
	Female	0	14	43	62		
Subject lecturers often do not turn up during the first 15 min of the examinations to answer student questions	Male	2	6	8	13	0.757	0.747
	Female	14	30	24	51		
Lecturers do not penalize late submission of assessment tasks	Male	2	4	10	13	0.228	0.210
	Female	14	30	22	53		
Some examination papers are too easy to assess learning outcomes at degree level	Male	0	10	12	7	0.398	0.443
	Female	0	56	36	27		
Most examination papers only test factual knowledge and not student understanding	Male	0	17	12	0	0.835	0.835
	Female	0	65	54	0		

At the level of significance of 0.05 there is no significant association or correlation between gender and the students perceptions or views agreements/disagreements about the practices and experiences by Chi-Square Test (p > 0.05).

Male and female students do not significantly differ in their views and perceptions agreement related to practices and experiences (p > 0.05) by the Fisher Exact Test.

Continuous assessment at the University of Namibia constitutes part of the assessment practices that ultimately determine whether or not the student will be promoted to the next year of studies. The researchers asked the respondents to indicate how long the lecturers took before returning the marked assignments to the students. The results showed that 55.4% of the students Agreed with the statement that their lecturers took too long in marking their assignments, while 44.6 disagreed. The mean score was 2.45 (95% Critical Interval: 2.37–2.53).

Students are always anxious to see how they have performed on their continuous assessment tasks (e.g., tests, assignments, etc.), what and how they did wrong, and how they might correct their mistakes will depend on the speed with which the lecturer provides this feedback. Therefore, it is important for the students to receive prompt feedback from their lecturers if this feedback is to be of benefit to them in enhancing their understanding of the subject content. The overall results showed that 66.2% of the students agreed that they received prompt feedback from their lecturers on assignments and tests (66.2%). The mean scores for these two items were 2.34 (95% Critical Interval: 2.26–2.42) and 2.34 (95% Critical Interval: 2.21–2.47). Thus, the results indicated that the majority of the students were of the opinion that lecturers gave prompt feedback on assignments and tests.

The researchers also wanted to know from the students as to whether lecturers gave helpful feedback on assignments as well as on tests. The students' views on these two items differed. Fifty five per cent agreed that the lecturers gave helpful feedback on assignments, 12.2% disagreed and 32.4% strongly disagreed with the statement. On the other hand 43.2% of the students agreed that the lecturers gave helpful feedback on tests, and 56.8% disagreed. The mean scores were 2.77 (95% Critical Interval: 2.62–2.92) and 2.57 (95% Critical Interval: 2.49–2.65) respectively. The results showed a split in views on the two items. Some students agreed that lecturers gave helpful feedback on assignments while others disagreed (see Table 2).

Due to sickness, sometimes some students miss tests. However, they still have to make up for the missed tests by taking the same or very similar test at a later time. In this study the researchers wanted to find out whether in the students' views being allowed to take a missed test presented an unfair advantage to those who had to sit for the same test at a later time when all other students had already written the same test. The results show that 33.8% of the students agreed, 44.6% disagreed and 21.6% strongly disagreed. The mean score for this statement was 2.54 (95% Critical Interval: 2.35–2.73). This result suggests that the majority of the students held the view that providing a second chance to students who had missed the test-on humanitarian reasons did not give unfair advantage to students who wrote the test later.

The findings reported in Table 2 are further supported by the results obtained from the calculations of the Fisher Exact Test and the Chi-Square test as shown in Table 3.

**Table 3:** **Provision of feedback to the students on assessment tasks**.

	***N***	**Minimum**	**Maximum**	**Score**		**95% confidence interval**
				**Mean**	**Std. error**	
My lecturers take a long time in marking assignments	148	2	3	2.45	0.041	2.37–2.53
My lecturers provide prompt (quick) feedback on assignments	148	2	3	2.34	0.039	2.26–2.42
My lecturers provide prompt (quick) feedback on written tests	148	1	4	2.34	0.067	2.21–2.47
My lecturers provide helpful feedback on assignments	148	2	4	2.77	0.075	2.62–2.92
My lecturers provide helpful feedback after the tests	148	2	3	2.57	0.041	2.49–2.65
Second opportunity tests gives unfair advantage to students who wrote the first test	148	1	4	2.54	0.096	2.35–2.73

The results show that at the 0.05 significant level, there was no significant difference for most of the items except for item 3, “My lecturers provide prompt (quick) feedback on written tests” between male and female students. This finding is also confirmed by the Fisher exact Test. It is interesting to note indeed the majority of the female students were of the view that their lecturers provided prompt feedback on their written work as compared to the male students.

### Final examinations conduct

Under the University of Namibia examinations, students have the right to appeal against the examination mark assigned to them by the lecturer. This often occurs when the mark is a failing one and their examination script is then remarked by another lecturer in the Department or Faculty. Accordingly, in this study we asked the students whether the final examinations appeal process was too long or not. The results showed that 10.8% strongly agreed, 45.9% agreed, 10.8% strongly disagreed and 32.4% disagreed. The mean score was 2.43 (95% Critical Interval: 2.30–2.56). The results suggest that the majority of the students agreed that the final examination appeal process was too long (See Table 3).

The clarity of questions on any test or examination question paper is of critical importance to how students respond to the questions asked. Ambiguity of questions can have serious implications to students' performance on the assessment tasks; therefore, lecturers have to make sure such ambiguity is minimized on assessment tasks. Asked whether examination questions were often not clearly stated, more students agreed that this indeed was the case. Close to 11% of the respondents strongly agreed, 33.8% agreed while 55.4% disagreed with the statement. The mean score was 2.45 (95% Critical Interval: 2.34–2.56), suggesting that the perceptions of the majority of the students was that examination questions were often clear.

As to whether the examination papers usually did not cover the content taught in class, a small number of the respondents agreed with the statement. Close to 11% of the students strongly agreed, 10.8% agreed, 56.8% disagreed, and 21.6% strongly disagreed. The mean score was 2.89 (95% Critical Interval: 2.75–3.03)(See Table 4). Thus, the results seem to show that the students believed that examination papers covered the subject content taught in class.

**Table 4:** **Continuous assessment feedback**.

	**Gender**	**Strongly agree**	**Agree**	**Disagree**	**Strongly disagree**	**Fisher Exact Test**	**Pearson Chi-Square Test**
						***p*-value**	***p*-value**
My lecturers take a long time in marking assignments	Male	0	17	12	0	0.835	0.835
	Female	0	65	54	0		
My lecturers provide prompt (quick) feedback on assignments	Male	0	20	9	0	0.828	0.828
	Female	0	78	41	0		
My lecturers provide prompt (quick) feedback on written tests	Male	0	20	4	5	0.032^*^	0.049^**^
	Female	16	62	30	11		
My lecturers provide helpful feedback on assignments	Male	0	20	2	7	0.336	0.270
	Female	0	62	16	41		
My lecturers provide helpful feedback after the tests	Male	0	16	13	0	0.209	0.209
	Female	0	48	71	0		
Second opportunity tests gives unfair advantage to students who wrote the first test	Male	7	0	18	4	0.135	0.116
	Female	43	0	48	28		

* Male and female students significantly differ in their views and perceptions agreement related to continuous assessment feedback (p < 0.05) by the Fisher Exact Test.

** At the level of significance of 0.05 there is a significant association or correlation between gender and the students perceptions or views agreements/disagreements about the continuous assessment feedback by Chi-Square Test (p < 0.05).

The leaking of examination questions to the students before the examinations is always a possibility that must be guarded against. The researchers wanted to find out from the students whether or not examination papers were kept in a safe place. Close to 46% of the students agreed, 21.6% agreed, and21.6% strongly disagreed) that examination papers were not kept in a safe place. For this statement a mean score of 2.76 (95% Critical Interval: 2.58– 2.94) was obtained.

To see whether the students would give consistent responses to a similar but differently stated question, the researchers also asked the students whether examination papers were often leaked to the students before the examinations actually took place. The results (Table 4) show a discrepancy in the views of the students between the first and the second item. A large number of the respondents disagreed (78.4%) while 31.6% agreed that the examination papers were leaked to the students before the examinations were written. The mean score for this item was 3.23 (95% Critical Interval: 3.07–3.39). This finding seems to suggest that not only were the students' views divided on whether examination papers were kept in a safe place, but they were also divided on whether the examination papers were leaked to the students before they were written. The possible reasons for this divided view on both items is probably due to the fact that, not all students would admit to the fact that the examination papers were not kept in a safe place or were leaked before the actual writing of the examination paper because this would have implications for the quality assurance of the whole assessment process and its validity to the outside world. Further, admitting that the examination papers were leaked would need to be substantiated, a prospect not appealing to the students.

With regard to whether the lecturers asked the same examination questions all the time, only 10.8% of the students agreed. A total of 89.2% (77% disagreed and 12. 2% strongly agreed) disagreed that the lecturers asked the same questions all the time. The mean score was 2.91 (95% Critical Interval: 2.79–3.03). Thus, this result indicates that a large majority of the students were not of the opinion that the lecturers asked the same questions all the time. If a lecturer asked the same questions all the time, the possibility of obtaining undeserved marks would be enhanced.

Generally, there are varied negatively expressed perceptions among University lecturers on the use of multiple choice examinations questions to assess University students. One of the reasons given for this pessimistic view is that multiple choice examinations do not allow assessment of the students' critical thinking and that it only assesses factual information or the lower levels of Bloom's cognitive domain. University lecturers teaching large classes (anything in excess of 100 students) view multiple choice examinations as their salvation when it comes to marking the students' scripts expediently, providing the all-important feedback to the students and meeting the set deadlines of submitting the examination results to the Registrar's office. Accordingly in this study, the students were asked whether the lecturers often used multiple choice questions in their examinations. The results revealed that a large number of the students (66.2%) agreed that this was indeed the case (21.6% strongly agreed and 44.6% agreed). Only 33.8% disagreed that lecturers often used multiple choice questions in the examinations. The mean score was 2.12 (95% Critical Interval: 2.00–2.24). It can therefore, be concluded that lecturers mainly used multiple choice questions in the examinations.

As to whether examination questions were usually of a short-answer nature, 54% of the students agreed (21.6% strongly agreed, 32.4% agreed) with the statement while 46% disagreed (33.8% disagree, 12.2% strongly disagreed). The mean score was 2.36 (95% Critical Interval: 2.21–2.51). Thus, the results suggest that more students believed that examination questions were of a short-answer nature. The responses to this statement if taken together with the responses on whether the lecturers usually asked multiple choice questions in the examinations, it can be deduced that the University of Namibia examination questions are a combination of multiple choice questions and short-answer questions.

In Table 5 we present the Fisher Exact Test and Chi-Square Test results for the eight items related to the respondents' views regarding the conduct of the final examinations.

**Table 5:** **Conduct of the final examination and the appeal process**.

	***N***	**Minimum**	**Maximum**	**Score**		**95% confidence interval**
				**Mean**	**Std. error**	
The final examination appeal process is too long	148	1	4	2.43	0.068	2.30–2.56
The examination questions are often not clearly stated	148	1	3	2.45	0.056	2.34–2.56
The examination papers usually do not cover the content taught in class	148	1	4	2.89	0.071	2.75–3.03
Examination papers are not kept in a safe place	132	1	4	2.76	0.094	2.58–2.94
Examination papers are often leaked to the students before the examination actually takes place	148	1	4	3.23	0.084	3.07–3.39
Lecturers ask the same examination questions all the time	148	1	4	2.91	0.061	2.79–3.03
My lecturers often use multiple choice questions in the examinations	148	1	3	2.12	0.061	2.00–2.24
Examination questions are usually of a short answer nature	148	1	4	2.36	0.079	2.21–2.51

The results in Table 5 render support to the results presented earlier in Table 4 in the sense that both the Fisher Exact Test and the Chi-Square Test show that at the 0.05 significance level there is no significant association between gender and the students perceptions about the Final Examinations by chi-square test (*p* > 0.05). Indeed, both male and female students do not significantly differ in their views and perceptions related to the Final Examinations.

### Administration of examinations

Administration of examinations is a complex and lengthy process and includes, actual planning for the examination, when it takes place, what subjects will be written and when. This planning involves, printing of examination papers, identification of venues, timetabling, of regular, supplementary and special examinations (where applicable), identifying the invigilators and delivery of examination papers to the right venues. One of the statements requested the respondents to indicate whether the examination papers were often misplaced at the examination office. Sixty seven per cent of the students disagreed that examination papers were often misplaced, while 32.4% agreed. The mean score was 2.91 (95% Critical Interval: 2.82–3.00). See Table 6.

**Table 6:** **Factors associated with the students' views and perceptions on final examinations**.

	**Gender**	**Strongly agree**	**Agree**	**Disagree**	**Strongly disagree**	**Fisher Exact Test**	**Pearson Chi-Square Test**
						***p*-value**	***p*-value**
The final examination appeal process is too long	Male	2	15	7	5	0.398	0.397
	Female	14	53	41	11		
The examination questions are often not clearly stated	Male	2	7	20		0.289	0.260
	Female	14	43	60			
The examination papers usually do not cover the content taught in class	Male	0	3	17	9	0.113	0.148
	Female	16	13	67	23		
Examination papers are not kept in a safe place	Male	2		15	8	0.091	0.111
	Female	30		53	24		
Examination papers are often leaked to the students before the examination actually takes place	Male	2	0	10	17	0.069	0.085
	Female	14	16	24	65		
Lecturers ask the same examination questions all the time	Male	2		25	2	0.513	0.454
	Female	14		89	16		
My lecturers often use multiple choice questions in the examinations	Male	5	11	13		0.380	0.366
	Female	27	55	37			
Examination questions are usually of a short answer nature	Male	7	9	11	2	0.801	0.779
	Female	25	39	39	16		

At the level of significance of 0.05 there is no significant association or correlation between gender and the students perceptions or views agreements/disagreements about the final examinations by Chi-Square Test (p > 0.05).

Male and female students do not significantly differ in their views and perceptions agreement related to the final examinations (p > 0.05) by the Fisher Exact Test.

**Table 7:** **Examination administration at the University of Namibia**.

	***N***	**Minimum**	**Maximum**	**Score**		**95% confidence interval**
				**Mean**	**Std. error**	
Examination papers are often misplaced at the examination office	148	1	4	2.91	0.098	2.82–3.00
Scheduled examination timetables are often changed at short notice	132	1	4	2.76	0.083	2.60–2.92
Cases of students who miss examinations are handled on an Ad Hoc basis	116	2	3	2.43	0.046	2.34–2.52
Special examinations place an undue burden on the lecturers	132	1	3	2.38	0.060	2.26–2.50
Examinations start on time	148	1	4	2.45	0.078	2.30–2.60

Sometimes, due to medical reasons and/or other emergencies, students are not able to sit for the examinations at the scheduled times. In such cases, where students can provide the necessary evidence for missing an examination, the University of Namibia allows them to sit for an alternative examination(s) (e.g., special examinations). The respondents were asked to indicate whether the case(s) of students who missed examinations were handled systematically or on an Ad-Hoc basis. According to the findings, 44.6% of the students agreed with the statement that these cases were handled on an Ad-Hoc basis, while 33.8% disagreed. For this statement the mean score was 2.43 (95% Critical Interval: 2.34–2.52) leaning towards the disagree rating. See Table 6.

As to whether special examinations put undue pressure on the lecturers, students expressed the view that this was true. This view is reflected in the results that showed that 66.2% of the students agreed (i.e., 10.8% strongly agreed and 55.4% agreed) with the statement, while only 33.8% disagreed (12.2% disagreed and 21.6% strongly disagreed). The mean score was 2.38 (95% Critical Interval: 2.26–2.50).

It is very important that scheduled examinations begin at the time they are scheduled to begin. The researchers wanted to gauge the perceptions the students held about the punctuality of the administration of the examinations. About 11% of the respondents strongly agreed that examinations started on time and 55.4% agreed. However, 12.2% disagreed and 21.6% strongly disagreed. The mean score for this item was 2.45(95% Critical Interval: 2.30–2.60), suggesting that overall, the majority of the students' perceptions were that scheduled examinations began on time. Nonetheless, as indicated earlier in this report at times misplaced examination papers resulted in the delay of the start of the examination or postponement to a later date. The majority of the Medical and Pharmacy students in this study do not seem to have experienced this problem so far.

### Medical and pharmacy students' views on the fairness of examinations at UNAM

Sometimes students feel that the marking of examination scripts was not fair. Hassel and Lourey (2005) note that University staff shouldn't give marks for the sake of making the students happy. Accordingly the researchers sought to assess how prevalent this view was among the respondents. Thus, when asked this question, 77.4% of the students disagreed (56.8% disagreed and 21.6% strongly disagreed) that the marking of scripts was unfair. Only 21.6% of the students expressed the view that the marking of examination scripts was unfair. The mean score of 3.00 (95%Critical Interval: 2.35–3.10) seems to support the view that the marking of examination scripts was fair in the University.

There has been a perception among the University academic community that the scheduling of special examinations is not always communicated promptly to the students needing such an examination. This results in some students either arriving late or missing these examinations completely. The researchers addressed this question by asking the respondents whether this was a widespread problem. The students' views on this item were evenly divided, with half of the students (44.6%) agreeing and the other half (44.6%) disagreeing that students usually missed special examinations due to communication problems. The mean score for this statement was 2.50 (95% Critical Interval: 2.35–2.65), indicating an almost neutral view on this item.

The minimum mark required to be admitted to any examination at the University of Namibia is a 40% mark and to pass a module one should obtain 50% which is a composite mark of Continuous Assessment and Final Examination mark. However, quite often borderline cases occur where students obtain a 49%, just 1% point short of the passing mark of 50%. In such cases, a student's mark may be “condoned” or raised to 50%, depending on circumstances (e.g., regular class attendance, etc.) to enable them pass the module. Some students believe that it is not always that this exercise is carried out with a high degree of fairness and that some students do not actually benefit from such “condoning.” Accordingly, the researchers asked the respondents to indicate their views on the issue of “condoning” a 49%-mark to a passing mark of 50%. For this item, about 22% of the students did not respond to this question. However, with 78% of them responding, the results showed that 56.8% agreed while 21.6% strongly disagreed that the condoning of examination marks from 49% to 50% to pass a module was carried out fairly. The mean score was 2.55 (95% Critical Interval: 2.39–2.71). The results for this item appear to confirm the perception that marks were not always fairly condoned from 49%–50%.

According the UNAM regulations (UNAM, 2011, p. 18–20) students who score between 45% and 49% in an examination automatically qualify for the supplementary examinations. This is another opportunity for students to obtain a minimum passing mark in a given module. The; highest score they could obtain in the supplementary examination being 50%. The researchers wanted to know from the students whether, in their view, all students who obtain between 45% and 49% in an examination are given the opportunity to sit for the supplementary examinations. The results show that the majority of the students (89.2%) agreed that all students who score between 45% and 49% were given a second chance to earn a passing mark, while only 10.8% disagreed. The mean score was 2.00 (95% Critical Interval: 1.87–2.13).

It is interesting to note that most of the Medical and Pharmacy students were of the view generally that the assessment with regard to the administration and conduct of examinations at the University was fairly carried out. This in itself is probably an indication of the importance assigned to the conduct of examination by the lecturers and the registrar's Office and the view to provide the students adequate opportunities to pass their modules so as to ensure completion of their degrees with the prescribed number of years.

### Impact on: motivation/morale, progression, graduation, expenses

Summative assessment of the students has both short-term and long-term impact on the students' progress, motivation, graduation and finances. Accordingly, poorly administered examinations negatively impact on the above aspects and undermine the quality of assessment at an institution. The results in this study show that 78.4% of the students were of the view that the examinations at the University of Namibia were poorly administered. Only 10.8% of the students had an opposing view. The mean score for this item was 1.52 (95% Critical Interval: 1.40–1.64). This result seems to suggest strongly that the University must always ensure that examinations are administered fairly and effectively.

If poor administration of examinations undermines the quality of assessment, missing examination papers is an even worse scenario because it wastes time, increases students' anxiety and increases the chances of examination leakages. The researchers asked the students to indicate their opinion on the question of whether missing examination papers increased their examination anxiety. All 148 respondents (100%) agreed that misplacement of examination papers increased their anxiety (see Table 8). For this statement the mean score was 1.34 (95% Critical Interval: 1.26–1.42). Given this response, the management of examination papers including student examination scripts should be handled with extreme care to avoid stressing the students further. They are already stressed by the mere fact they have to sit for an examination that potentially determines their future employment prospects.

**Table 8:** **Expressed views on the fairness in the conduct of examination at the University of Namibia**.

	***N***	**Minimum**	**Maximum**	**Score**		**95% confidence interval**
				**Mean**	**Std. error**	
The marking of examination scripts is not fair	148	2	4	3.00	0.054	2.35–3.10
Usually students miss special examinations because of communication problems	132	1	4	2.50	0.075	2.35–2.65
The condoning of examination marks from 49% to 50% to pass is carried out fairly	116	2	4	2.55	0.083	2.39–2.71
All students who obtain between 45% to 49% in an examination are given the opportunity to sit for supplementary examination	148	1	4	2.00	0.066	1.87–2.13

Due to a variety of reasons, examination timetables may sometimes be changed at short notice. But, this should be on very rare occasions and should be avoided at all costs; since it increases the students' examination anxiety and puts in disarray their prior study and planning. The researchers therefore wanted to find out how sudden timetable changes affect the respondents' stress levels and performance on the examinations. The results indicate that 89.1% of the students agreed that changes in examination timetables put stress on them, while only 10.8% disagreed. Similarly, 89.2% of the students also reported that changes of examination timetable affected their performance negatively. The respective mean scores for these two items were 1.68 (95% Critical Interval: 1.57–1.79) and 1.78 (95% Critical Interval: 1.68–1.88).

Repetition of failed examinations papers is a very common exercise in universities across the world (Rom and Musgrave)). Unfortunately, such a situation might have additional financial implications for the student, family and those who fund their studies. In this study the respondents were asked whether repeating failed examination papers placed unnecessary financial burden on them and their families. Over 78% of the students agreed (21.6% strongly agreed and 56.8% agreed). Only 21.6% disagreed. The mean score for this item was 2.00 (95% Critical Interval: 1.89–2.11). See Table 6.

If chaos prevails in the administration of examinations and the marking of the students' scripts, many adverse effects are possible. Students may blame the University and the lecturers if they fail to graduate in time, particularly if the University is negligent in its conduct of the examinations or at fault in one way or the other. The researchers asked the students whether some students failed to graduate because of missing examination papers or because the timetable was changed at short notice. The results (See Table 8) show that 44.6% of the students agreed and 33.8% disagreed with the statement. The mean score was2.43 (95% Critical Interval: 2.27–2.59). This finding suggests that the University and the lecturers should be careful not to disadvantage the students unnecessarily.

The University of Namibia's regulations UNAM (2011, p. 20–25) state that if a student fails the same course/module more than three times, he/she cannot be allowed to continue taking that course/module. This regulation can have negative implications to a student's academic progress, graduation and financial independence later. The researchers therefore, asked the students whether they agreed with the statement that students fail to graduate because they repeatedly failed one module. Surprisingly, 22% of the students did not respond to this item. The results revealed an even split in the opinions of the students. About 45% of the respondents agreed and 43.8% disagreed with the statement. A mean score of 2.29 (95% Critical Interval: 2.10–2.48). This result seems to suggest that some students might not have been aware of the regulation governing repeated failure of modules at the University and the implications it might have on their progress and graduation.

### Ethical conduct of examinations

The researchers wanted to know from the students whether lecturers at the University of Namibia conducted themselves with integrity when invigilating examinations. The findings revealed that all the students agreed (56.8% agreed and 43% strongly agreed) that the lecturers carried out their invigilation duties with integrity. The mean score was 1.57 (95% Critical Interval: 1.50–1.65). The University of Namibia has a clear policy UNAM (2011, p. 12) regarding academic dishonesty. All students are expected to be familiar with this policy and adhere to it because cheating on tests, examinations, and other assessment tasks can have severe consequences for the student. Asked whether students often copied each other's work, 77% of the students disagreed with the statement, while 23% agreed with the statement. The mean score was 2.88 (95% Critical Interval: 2.74–3.02)(See Table 9).

**Table 9:** **Impact of assessment practices on motivation/morale, progression, graduation, expenses**.

	***N***	**Minimum**	**Maximum**	**Score**		**95% confidence interval**
				**Mean**	**Std. error**	
Poor administration of examinations undermines the quality of assessment	132	1	3	1.52	0.061	1.40–1.64
Missing examination papers increase students' anxiety	148	1	2	1.34	0.039	1.26–1.42
Change of examination timetables puts stress on the students	148	1	3	1.68	0.054	1.57–1.79
Change of examination timetable affects the performance of students negatively	148	1	3	1.78	0.051	1.68–1.88
Repeating failed examination papers places unnecessary financial burden on the students and their families	148	1	3	2.00	0.054	1.89–2.11
Some students fail to graduate because of missing examination papers whose timetable were changed at short notice	116	1	4	2.43	0.083	2.27–2.59
Students fail to graduate because they repeatedly fail one module	116	1	4	2.29	0.095	2.10–2.48

Another item regarding students' academic dishonesty gauged the students' views on whether the lecturers penalized students who copied other people's work. Plagiarism is regarded a serious case at the University of Namibia, since it involves the use of another person's intellectual ideas without being acknowledgement. The findings in this study show that, 55% of the students believed that lecturers do penalize students who copy other people's work, while 33.8% were of the view that the lecturers did not penalize students who plagiarized other people's work. The mean score was 3.00 (95% Critical Interval: 2.81–3.19).

The lecturers' biases in grading students' assignment is sometimes encountered in academic settings (Mark, 2013). In such a situation some lecturer's pet students may be assigned an undeserved higher grade, which might demoralize the hard working students. It should be pointed out that students (in the experiences of the researchers) tend to compare their work with each other and know work of high quality, mediocre and average and as such tend to know when a student is being favored by the lecturer. Therefore one of the items on the questionnaire asked the respondents to indicate whether in their view the lecturers at the University of Namibia were impartial in the grading of students' assignments. The results showed that 66.7% of the students were of the view that the lecturers were impartial in their marking of the students' work. This to us again shows that the lecturers showed greater integrity in their conduct of assessment in their subjects. The mean score was 2.76 (95% Critical Interval: 2.58–2.94) (see Table 8).

As far as the results on the ethical conduct of the assessment process at the University of Namibia is concerned, it seemed the lecturers displayed exemplary conduct by being impartial in their conduct and ensuring that all students got the deserved scores and grades. This behavior could be regarded as a hallmark of quality assurance in the conduct of examinations.

### Effects of gender on students' views and perceptions on assessment practices and experience

The Fisher Exact Test and the Pearson Chi-Square Test we carried out on the 13 items reflecting the students' perceptions on practices and experiences of the conduct of assessment at UNAM (See Table 10).

**Table 10:** **Ethical conduct of examinations**.

	***N***	**Minimum**	**Maximum**	**Score**		**95% confidence interval**
				**Mean**	**Std. error**	
Lecturers conduct themselves with integrity when invigilating examinations	148	1	2	1.57	0.041	1.50–1.65
Students often copy each other's work	148	1	4	2.88	0.072	2.74–3.02
Lecturers do not penalize students who copy other people's work	132	1	4	3.00	0.097	2.81–3.19
The grading of assignments is often partial (i.e., high marks are given to the students the lecturer likes)	148	1	4	2.76	0.093	2.58–2.94
Some lecturers give high marks to avoid being queried by the students	148	1	4	3.09	0.082	2.93–3.25
Some lecturers give high marks to ensure that they achieve high pass rates to show that they are better than other lecturers	148	1	4	3.09	0.082	2.93–3.25
To be popular with students, some lecturers award undeserved high marks	148	2	4	3.22	0.076	3.07–3.37

Table 9 shows that at the 0.05 level of significance there was no significant difference between gender and the students' perceptions on the practices and experiences as far assessment at the University was concerned. Both tests showed no significant difference was found at this level. This seems to indicate that both sexes perceived the practices and experienced of assessment in a similar light.

## Conclusion

The results in this study seem to point to an assessment process that is geared towards the attainment of quality in its practice and conduct (Airasian, 1997). Nonetheless, the administration of the assessments in some cases needs to be improved as perceived by the students in the Medical and Pharmacy schools. The students' perceptions of the fact that lecturers were impartial in their marking of students' work is encouraging, even though unsubstantiated media reports had painted a picture of an examination process that was beset by problems.

Examinations are a light that reflects the quality of staff and enhances the credibility of an institution. The students' positive perceptions of the conduct and practice of examinations in this study is an indication that regardless of the identified shortcomings, the students are fairly assessed at the Schools of Medicine and Pharmacy. Furthermore, there does not seem to be significant differences in the responses of both male and female respondents on the conduct of the assessment process at the University of Namibia, expect on the item referring to the provision of prompt feedback on written tests, which is significant at the 0.05 for both the Fisher Exact Test and the Pearson Chi-Square Test. As far as the conduct of the final examinations is concerned there was no significant difference between males and females as the 0.05 significance level using the Fisher Exact Test and the Chi-Square Test. In our view these results seem to suggest that both males and females experienced the conduct of the assessment process at the University of Namibia in a similar manner.

This study was limited by the fact that it only surveyed Medical and Pharmacy students, in the recently established Schools. The respondents in this study were not representative of the student body at the University of Namibia as they constituted a small fraction of that body. Because of this, it is possible that their views might beat variance with students from other Faculties in the University with large numbers of students. To address this limitation, an analytical study including all Schools and Faculties at different satellite campuses and at different levels of study in the different programmes needs to be carried out.

### Conflict of interest statement

The authors declare that the research was conducted in the absence of any commercial or financial relationships that could be construed as a potential conflict of interest.

## References

[B1] Airasian P. W. (1997). Classroom Assessment. 3rd Edn New York, NY: McGraw-Hill

[B2] Brown S. Knight P. (1994). Assessing Learners in Higher Education. London: Kogan Page Ltd

[B3] Grunwald H. Peterson M. W. (2003). Factors that promote faculty involvement in and satisfaction with institutional and classroom student assessment. Res. High. Edu. 44, 173–204

[B4] Hassel H. Lourey J. (2005). The dea(r)th of student responsibility. College Teach. 53, 2–13 10.3200/CTCH.53.1.2-13

[B5a] Mark C. R. (2013). Student and Instructor Assessment: From Dark Ages to Utopian Future, (Georgetown University), 1–12 Available online at: http://wiscape.wisc.edu/uploads/media/5cf45fd2-c49a-40cc-8af2-30f614dc82c5.pdf (last accessed January 12, 2013).

[B5] McMillan J. H. Myran S. Workman D. (2002). elementary teachers' classroom assessment and grading practices. J. Edu. Res. 95, 203–213 10.1080/00220670209596593

[B6] Miller M. D. Linn L. Gronlund N. E. (2012). Measurement and Assessment in Teaching, 11th Edn Melbourne: Pearson Australia Group

[B7] Morgan C. Watson A. (2002). The interpretative nature of teachers' assessment of students' mathematics: issues for equity. J. Res. Math. Edu. 33, 78–110 10.2307/749645

[B8a] Njabili A. F. Kasanda C. D. (2005). Education in Namibia - A Collection of Essays. Namibia: University of Namibia Publishers (ISBN: 99916-67-68-7)

[B8] Rom M. C. Musgrave P. (nd). Political Bias in Grading: Identifying Problems, Suggestions Solutions. Georgetown University. Available online at: https://www.goacta.org/publications/downloads/ChurchillFinal.pdf, access on 27/03/2013

[B9] Saliu S. (2005). Constrained subjective assessment of student learning. J. Sci. Edu. Technol. 14, 271–284 10.1007/s10956-005-7193-1

[B10] Santos J. R. A. (1999). Cronbach's alpha: a tool for assessing the reliability of scales. J. Exten. 37, 34–36

[B11] Tavakol M. Dennick R. (2011). Making sense of Cronback's alpha. Int. J. Med. Edu. 2, 53–55 10.5116/ijme.4dfb.8dfd 28029643 PMC4205511

[B12] UNAM. (2011). General Information and Regulations Prospectus 2012, Windhoek: University of Namibia

[B13] Zimba R. F. (2005). Authentic University Learning, Teaching and Assessment: A Professorial Inaugural Lecture. Windhoek: University of Namibia Press

